# Considerations for Community-Based mHealth Initiatives: Insights From Three Beacon Communities

**DOI:** 10.2196/jmir.2803

**Published:** 2013-10-15

**Authors:** Nebeyou A Abebe, Korey L Capozza, Terrisca R Des Jardins, David A Kulick, Alison L Rein, Abigail A Schachter, Scott A Turske

**Affiliations:** ^1^Louisiana Public Health InstituteCrescent City Beacon CommunityNew Orleans, LAUnited States; ^2^HealthInsightUtah Beacon CommunitySalt Lake City, UTUnited States; ^3^Southeast Michigan Beacon CommunityDetroit, MIUnited States; ^4^AcademyHealthWashington, DCUnited States

**Keywords:** mHealth, mobile health, mobile phone, Type 2 diabetes mellitus, text messaging, short message service (SMS), risk reduction, self management

## Abstract

Mobile health (mHealth) is gaining widespread attention for its potential to engage patients in their health and health care in their daily lives. Emerging evidence suggests that mHealth interventions can be used effectively to support behavior change, but numerous challenges remain when implementing these programs at the community level. This paper provides an overview of considerations when implementing community-based mHealth initiatives, based on the experiences of three Beacon Communities across the United States that have launched text messaging (short message service, SMS) pilot programs aimed at diabetes risk reduction and disease management. The paper addresses lessons learned and suggests strategies to overcome challenges related to developing text message content, conducting marketing and outreach, enrolling participants, engaging providers, evaluating program effectiveness, and sustaining and scaling the programs.

## Introduction

Mobile health (mHealth), defined as “medical and public health practice supported by mobile devices, such as mobile phones, patient monitoring devices, personal digital assistants, and other wireless devices,” is increasingly used to engage patients in their health and care [1]. Cell phone use is widespread across socioeconomic, racial, ethnic, and age groups; 91% of Americans use cell phones, and 80% of cell phone users engage in text messaging (short message service, SMS) [2]. Additionally, Hispanic and black Americans—who are often medically underserved—are more likely to use text messaging than white Americans (85% vs 80% vs 79%, respectively) [3]. The near-ubiquity of cell phones, and their use for texting, demonstrates the potential of text message-based mHealth programs to reach traditionally underserved and hard-to-reach populations.

A growing body of evidence supports the feasibility of using text messaging and other mHealth applications for health promotion [4-6], behavior change (eg, smoking cessation) [7-10], chronic disease management [11], medication adherence [12,13], prenatal care [14,15], weight loss [16,17], and physical activity [18-20]. These programs target health behaviors by providing information and services—including medical appointment and medication reminders, self-tracking tools, educational resources, lab and clinical results delivery, etc—via timely and often personalized messages [5]. Such mHealth services confer advantages over traditional informational public health campaigns by providing a medium for broader audience engagement and direct interaction.

While using mobile technology as a source of health information is a relatively new concept, recent studies suggest that patients are generally open to receiving health-related text and electronic messages [21,22]. Furthermore, 31% of cell phone owners report using their phone to look for health information in 2012, compared to only 17% in 2010 [23]. This growing appetite for receiving and seeking health information via mobile technology presents new opportunities to engage patients outside of traditional care settings, even those who do not regularly seek health care services.

Recognizing this potential, and intrigued by the opportunity to help manage and possibly prevent chronic disease, several communities across the United States receiving federal funding through the Beacon Community Cooperative Agreement Program have deployed mHealth programs. Full results of the Beacon Communities’ evaluation efforts are still pending, but early findings suggest a promising impact of mHealth on behavior change. However, these community-based pilots encountered a number of challenges in the design (eg, developing content; conducting outreach), execution (eg, engaging patients and providers), evaluation, and sustainability of their mHealth programs. The paper also describes lessons learned and offers strategies and promising practices to address these challenges (Table 1).

**Table 1:** Challenges, considerations, and lessons learned for developing community-based text-messaging programs.

Domain	Challenges / Considerations	Lessons Learned
**Developing message content**		
	Technical constraints (160 character limit)	Use evidence when developing messages
	Cultural competency / language availability	Tailor messages based on participant data
	Clinical validity / guidelines	Customize messages to local population to enhance user experience
	Literacy level testing	
	Tailoring to local population and individual users	
	Framing messages based on behavior change theories	
**Outreach and marketing**		
	Selecting media and outreach channels	Engage non-traditional partners
	Events vs marketing	Design a flexible outreach plan
	Targeted vs mass-media marketing	Enrollment proposition is more compelling in health-related contexts (eg, health fair vs sporting event)
**Enrolling participants**		
	Enrollment method impacts enrollment numbers and engagement	Offer multiple enrollment method options
	Limited technological proficiency and access	High-touch, in-person recruitment is key, but is labor intensive and costly
	Costs of participating/texting	
**Engaging providers**		
	Lack of payment under fee-for-service	Credibility drives adoption
	Lack of interoperability/data sharing between mHealth platforms and EHRs	Integrate mHealth into other interventions/initiatives
	Multiple and competing priorities	
**Evaluating impact**		
	Lack of robust mHealth evaluation methodologies	Plan evaluation strategy, identify data sources and outcome metrics from outset of project
	Limited funding	Minimize biases
	Accelerated timeline	Consider level of rigor needed and budget or other resource limitations
	Rigor / quality vs speed / cost	
	Biases (attrition, sampling, non-response)	
**Sustaining and scaling**		
	Sustaining programs after grant funding ends	Leverage community partnerships for financial and in-kind support
	Lack of provider reimbursement for mHealth	Partner with health plans
	Securing partnerships and resources	Incorporate mHealth into other payment reform strategies

## About the Programs

The 2009 Health Information Technology for Economic and Clinical Health (HITECH) Act authorized the Office of the National Coordinator for Health Information Technology to create the Beacon Community Cooperative Agreement Program, which granted 17 diverse communities across the United States $12-15 million each over 3 years to build and strengthen their health information technology (health IT) infrastructures and to test innovative technologies to improve care quality and population health and reduce costs [24]. Several of the Beacon Communities launched community-based mHealth programs as part of their health IT-enabled quality improvement efforts.

The Crescent City and Southeast Michigan Beacon Communities, located in and around New Orleans, Louisiana, and Detroit, Michigan, respectively, were two of three Beacon Communities (the Greater Cincinnati Beacon Community in Ohio was the third) to pilot txt4health**,** a text message–based health information service that aimed to help adults ages 18 and up to understand their risk of developing type 2 diabetes and steps they can take to reduce that risk. txt4health targeted highly vulnerable, at-risk populations in these communities, many of whom were overweight or obese, low-income, and/or uninsured. Participants enrolled in txt4health by texting the word “health” to 300400 or online via the txt4health website. Upon enrollment, participants completed a diabetes risk assessment, the results of which were used to place them in a risk category and to tailor subsequent text messages. Participants received 4-7 messages per week, including general educational messages, diet and exercise tips, health reminders, and information about local health care providers and resources. Participants could also set and track progress toward weight loss and exercise goals by responding to text message prompts.

The Utah Beacon Community, located in and around Salt Lake City, Utah, launched Care4Life**,** a two-way text messaging program designed to enhance self-management among adults aged 18 and up with type 2 diabetes. Care4Life participants received 1-7 messages per day over a period of 6 months. Like txt4health, Care4Life messages included general diabetes education, health improvement suggestions, and encouragement toward self-entered weight loss and exercise goals. In addition, Care4Life included robust coaching and interactive support functionality; participants could elect to receive medication, blood sugar testing, blood pressure monitoring, and clinical appointment reminders, as well as requests to report back medication adherence, blood sugar readings, physical activity, and weight. As with txt4health, Care4Life enrollees could join via text message or online; however, participants who enrolled by text received one-way educational messages only—unless they signed up for more protocols at a later date—whereas those who enrolled online received the full suite of two-way message options. Participants could also track their data via a Care4Life Web portal. See Table 2 for a comparison of txt4health and Care4Life.

**Table 2:** txt4health and Care4Life program characteristics.

	txt4health	Care4Life
Beacon Communities implementing	Crescent City and Southeast Michigan	Utah
Target population	Adults age 18+ at risk for type 2 diabetes	Adults aged 18+ diagnosed with type 2 diabetes with HbA1c>8^a^
Message types	Diabetes risk assessment	Diabetes education and health improvement
	General diabetes education	Medication, glucose testing, blood pressure monitoring, and clinical appointment reminders
	Diet and exercise tips	Encouragement toward self-entered weight loss and exercise goals
	Health reminders	Requests to report back blood sugar readings, medication adherence, exercise and weight loss goals
	Ability to set and track personal weight loss and exercise goals	
	Information about how to find local providers and resources	
Program duration	14 weeks	26 weeks
Message frequency	4-7 per week	7-49 per week

^a^HbA1c=hemoglobin A1c, a measure of blood sugar control.

## Program Planning: Developing Message Content

For the Crescent City and Southeast Michigan Beacon Communities, developing txt4health text message content involved more than simply adhering to character limits and considering literacy levels. The messages needed to contain clinically valid health information, presented in a tailored way that would appeal to the target population and ultimately promote behavior change.

To ensure clinical validity, the txt4health messages were developed by an advisory group that included members of the Crescent City, Southeast Michigan, and Cincinnati Beacon teams, as well as experts from the txt4health mHealth vendor (Voxiva), the Centers for Disease Control and Prevention, and the American Diabetes Association. The clinical content was based on evidence-based guidelines, including those endorsed by the American Diabetes Association (ADA) and the National Diabetes Education Program (NDEP); for instance, the risk assessment was used to assign txt4health participants into risk categories developed by the ADA, in order to tailor the text messages to the appropriate risk level. To vet their messages further, the Crescent City Beacon conducted focus groups and in-depth interviews with local providers and community leaders, as well as workgroups with representatives from consumer organizations including the Juvenile Diabetes Research Foundation.

**Figure 1:**
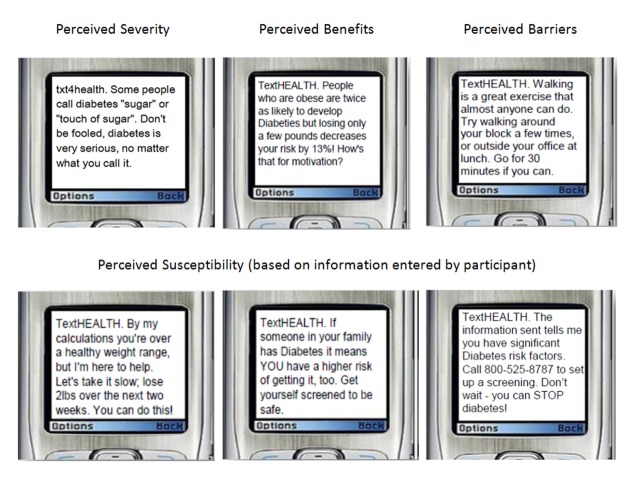
Sample txt4health messages and relevant Health Belief Model dimension.

The advisory group also drew from relevant research to formulate messages based on theories of behavior change, especially the Health Belief Model, a conceptual model describing factors that influence whether people engage in health behaviors such as preventive care or adherence to treatment regimens [25]. According to the model, the likelihood of engaging in a health behavior is influenced by one’s perceived susceptibility to a particular disease or condition, the perceived seriousness or severity of that condition, the perceived benefits of the behavior, and the perceived barriers to engaging in that behavior [25]. The communities deploying txt4health observed a lack of perceived risk of developing diabetes among potential enrollees, either in the form of real or perceived apathy toward the risk factors of diabetes, or in a lack of understanding of how risk factors affect the onset of diabetes. To address these perceptions, the advisory group crafted educational messages and recommendations based on the Health Belief Model, emphasizing participants’ susceptibility to diabetes (based on their risk assessment); the potentially severe consequences of developing or failing to control diabetes; the simple steps that can be taken to reduce one’s risk; and the short- and long-term benefits of those steps. See Figure 1 for sample messages that address each dimension of the HBM.

To appeal to txt4health’s broad target population, which included many people who did not regularly access health care, the Crescent City and Southeast Michigan Beacon teams also endeavored to the craft messages in an approachable, encouraging, and friendly “voice”. Furthermore, the messages aimed to be culturally competent, reflecting an understanding of local interpretations of disease and the colloquial language used to describe it. For example, in Southeast Michigan diabetes is often referred as “sugar”. By incorporating this term into the text messages, the txt4health team hoped participants would perceive the program as more accessible and relatable and would thus engage with it more actively. They also subjected the messages to literacy level testing to ensure that they were straightforward, easily understandable, and did not contain medical jargon.

The Crescent City and Southeast Michigan teams also created community-specific messages to reflect the local context. About once per week, both communities sent messages via txt4health to notify participants about upcoming local events such as health fairs. Additionally, recognizing that personal safety could be a concern in Detroit and the surrounding cities, the Southeast Michigan team included alternatives to outdoor activities (eg, renting an exercise video or exercising while watching TV) in their txt4health messages.

**Figure 2:**
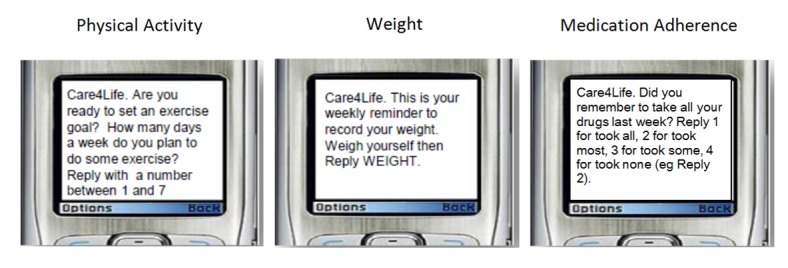
Sample Care4Life messages.

In contrast to the experience with txt4health, the Care4Life messages used in Utah were previously developed by a diabetes education expert, based on the ADA clinical guidelines, the NDEP, and principles of the American Association of Diabetes Educators AADE7 Self-Care Behaviors. The Utah Beacon team decided to use these pre-developed messages because they had already undergone a rigorous vetting process and were based on established clinical guidelines—an important feature given that the intervention was embedded in a clinical environment [26]. To preserve the validity of the messages, the text was not modified or customized for the Utah Beacon population; however, participants could select the types and frequency of messages received (eg, medication reminders, weight loss tracking, blood sugar testing requests), and the messages addressed participants by name. See Figure 2 for sample Care4Life messages.

Research suggests that framing health-related messages to target beliefs, perceptions, and subjective norms can influence their impact on attitudes and intentions and ultimately encourage behavior change [27-31]. While the txt4health teams used the Health Belief Model to target perceptions and beliefs, future iterations of both txt4health and Care4Life could evaluate how further tailoring and framing the messages based on user demographics or health risk assessment data could enhance user experience and the programs’ impact on targeted health behaviors.

## Outreach and Marketing

### Overview

All three Beacon Communities engaged in marketing and outreach in order to drive interest and enrollment in their mHealth programs. This engagement involved experimentation with different partners, media, and outreach strategies (Table 3), as generating the desired levels of enrollment proved challenging.

Outreach efforts in the Utah Beacon Community targeted patients treated at the 19 primary care clinics participating in a community-wide quality improvement effort around diabetes care. The outreach process was dictated by what clinic staff were willing and able to take on in terms of workload and provision of access to patient data. Since the pilot was conducted as a randomized controlled trial, outreach was also limited by human subjects research protocols. Clinic staff queried the clinics’ electronic health record (EHR) systems to identify patients with type 2 diabetes who might benefit from Care4Life and mailed invitations that directed them to sign up online. However, finding that many patients had difficulty with online enrollment, about 4 months into the recruitment efforts the Beacon team shifted to a more hands-on, in-person approach where staff stationed in clinics assisted patients with enrollment and offered training on how to navigate the Care4life program.

In contrast to Care4Life, txt4health targeted populations at risk for or with undiagnosed diabetes, whether or not they sought or accessed care. Therefore, while the Crescent City and Southeast Michigan Beacons supplied marketing materials to promote txt4health in primary care clinics, they also undertook comprehensive, multipronged marketing campaigns via a wide variety of outreach channels and settings. These channels included mass-media marketing through traditional vehicles such as television and radio, as well as social media and online advertising.

The Crescent City and Southeast Michigan Beacons were challenged in reaching a typically hard-to-reach target audience. With the support and resources from a leading national advertising and marketing firm, the Southeast Michigan Beacon drew from third-party syndicated research to identify areas in the Detroit area likely to have a high density of diabetic and pre-diabetic individuals and targeted outreach activity in these areas. They advertised txt4health in public transit, bus shelters, laundromats, barbershops, salons, and other settings frequented by the target audience, which allowed them to optimize outreach while conserving limited resources.

In Crescent City, the Beacon Community Advisory Group was instrumental in devising the txt4health outreach strategy, conducting focus groups and key informant interviews with consumers, providers, and other community members to better understand how to reach the target audience. The Crescent City team worked closely to leverage the advisory group members’ communication channels (eg, health fairs, church meetings, retail stores) for outreach efforts. For example, a large retail partner allowed txt4health staff to directly engage customers through in-store activations, and pharmacists working at this retail chain promoted txt4health directly to customers at risk for diabetes. Additionally, Crescent City engaged non-traditional partners such as faith-based organizations and university student groups to host enrollment contests and events. Crescent City relied less heavily on online marketing than Southeast Michigan, instead asking community partners to include txt4health information on their websites.

Having a flexible outreach plan allowed all three communities to change their tactics based on their experiences with various strategies. They also found that community partnerships, health fairs, and public events represented key opportunities to engage potential txt4health and Care4Life participants. In Crescent City and Southeast Michigan, in addition to driving enrollment, these partnerships and events also helped garner support and goodwill toward txt4health and the broader Beacon Community initiative.

**Table 3:** mHealth outreach and marketing channels and tactics.

Outreach Channel	Tactic	Beacon Communities Using		
		Crescent City	Southeast Michigan	Utah
Mass media	Television and radio public service announcements, (paid) radio advertising, “flash mobs,” online advertising, transit media (bus exteriors and interiors), in-place media (targeted signage), and earned media.	X	X	
Electronic marketing	Facebook, online advertising.	X	X	
Events	Community events, entertainment venues, health fairs, screenings.	X	X	X
Community partner marketing	Community partner events, websites, and newsletters.	X	X	X
Beacon interventions	Integrating mHealth program into other Beacon interventions (eg, Patient Health Navigator program; Emergency Department Diabetic Patient Identification program; diabetes quality improvement initiative).		X	X
Primary care practices	Directly involving primary care providers in promoting the program to patients and visitors	X	X	X
	Displaying marketing materials in exam and waiting rooms (eg, table tents, “prescription-like” tear-pads, posters).	X	X	X
Mass mailings	Using clinic data to identify patients likely to benefit from the program and mailing them an invitation with instructions on how to enroll.			X

### Enrolling Participants

As noted above, these outreach and marketing campaigns were designed to drive enrollment in txt4health and Care4Life. Participants could enroll in one of three ways (Table 4). The Beacon Communities found that the method used to enroll interested participants had an important influence on the total number of enrollees, as well as their subsequent level of engagement with the program.

In the case of txt4health, enrolling via text allowed potential participants to immediately “opt-in” to the service and proceed to the health risk assessment and subsequent messaging curriculum. Participants with a computer and Internet access could enroll online via the txt4health website and confirm their enrollment by responding to a confirmation text message triggered by data entered in the online form. Like text-based enrollment, this method required proactive participation on the part of the user, which translated to a higher likelihood of program “activation” upon receipt of the confirmation text message.

Third-party enrollment was used at health fairs and community events where Beacon staff promoted txt4health. At these events, potential enrollees provided their name, cell phone number, and ZIP code on a roster that included a consent waiver. After the event, Beacon staff would enter this information into the online enrollment form, triggering confirmation text messages for participants to respond to in order to activate their enrollment. The Beacon teams observed a significant drop-off in txt4health participant activation among those who were enrolled by a third party, which they attribute to many potential factors, including the time lag between initial sign-up and confirmation of participation in the program; the provision of incorrect or incomplete contact information on the roster; and/or the lack of direct personal participation in the enrollment process.

For Care4Life, the mode of enrollment had implications for program experience once enrolled. Those who enrolled by text message received one-way educational messages only but could add more message types via the Web portal or via text message at a later date. Those who enrolled online could receive the full suite of two-way message options including reminders, coaching, and requests for feedback. Additional information was required for online enrollment, including a series of health-related questions designed to set personalized reminder and coaching messages. This full enrollment process was more time consuming for patients to complete via text message than via a Web-based form.

To address this challenge, Beacon staff in participating clinics were equipped with computers and made available to help patients enroll in Care4Life online. Using this method, they signed up more than 400 patients in the program over a 6-month period. One clinic in Utah adopted a more aggressive approach, incorporating the Care4Life outreach and enrollment process directly into existing workflows. During regularly scheduled outreach calls to diabetic patients, medical assistants scheduled the quarterly recommended office visit and then signed interested patients up for Care4Life during the same call. Using this method, the clinic enrolled more than 40 patients in a period of 2 weeks, which represented a significant boost in enrollment.

Based on their experiences, these three Beacon Communities identified some key barriers to enrollment. First, although the txt4health and Care4Life programs were free, messaging rates applied for participants without unlimited texting plans, which proved cost-prohibitive for many potential participants who did not want to use up their limited messages. The txt4health teams identified this issue prior to the program launch, since at that time many people in the target population used government-issued cell phones whose service plans did not include unlimited texting. Unfortunately, it was not possible to assess how many potential participants were deterred from enrolling due to cost.

Limited technology proficiency and/or access to computers or the Internet presented additional barriers to online and text-based enrollment. During a follow-up telephone survey of 104 patients invited to participate in Care4Life, 35% reported limited or no access to a computer and 38% reported having trouble using a Web browser. Certain patient demographic characteristics were associated with lower technological proficiency; for instance, while older patients (age 50+) represented the majority of txt4health and Care4Life users, this group tended to struggle with using texting more than their younger counterparts. Many Care4Life participants also had very basic or older phones with more outdated features (eg, numerical keys rather than keyboard) that made texting more difficult, especially for older users. To address this challenge, Beacon staff in Utah and New Orleans trained Care4Life and txt4health participants, respectively, to send and receive text messages during in-person enrollment events.

All three Beacon Communities found that potential participants often needed the assurance of in-person interaction and personal relationship (eg, with a trusted provider) to get involved with these new, novel programs. While surveys indicated that the traditional marketing tactics such as advertisements and brochures increased community awareness of the programs, direct in-person engagement drove enrollment to a much greater extent. People were much more receptive to the txt4health and Care4Life “enrollment proposition” when they were open to or seeking health information. Whether at community events or in the clinic, additional staff support was critical to deliver the high-touch, one-on-one personal interactions and drive enrollment in the programs. However, this made the process much more labor-intensive and costly than anticipated. Other communities launching similar mHealth programs may learn from this experience by anticipating and budgeting for additional staff time and resources to support in-person enrollment efforts.

**Table 4:** Enrollment methods for txt4health and Care4Life.

Enrollment method	txt4health	Care4Life
Text	Text the word “health” to 300400	Text a unique, clinic-specific enrollment code to 300400
Online	Enter cell phone number and ZIP code in online enrollment form, then respond to confirmation text message triggered by the form	Complete online enrollment form (up to 26 questions), then respond to confirmation text message triggered by the form
Third party	Allow third party to complete online enrollment on behalf of user. Enrollee must respond to confirmation text message triggered by the online form.	Allow third party to complete online enrollment on behalf of user. Enrollee must respond to confirmation text message triggered by the online form.

## Engaging Providers

Based on the experiences of these three Beacon Communities, integrating mHealth programs in the clinical setting has several advantages. As anticipated, the Beacons found that introducing the programs in the health-related or clinical settings (rather than via mass media or community events) led to higher rates of adoption and greater credibility among participants. Unfortunately, several barriers may constrain further integration of mHealth into the clinical setting.

Two primary barriers to engaging clinicians and care teams in mHealth are related to the predominant fee-for-service payment structure. First, the current structure rewards patient volume, which limits appointment times—typically to 8-10 minutes. Second, providers are reimbursed only for specific activities, which do not generally include discussion or promotion of mHealth programs [32]. As such, while all three Beacon Communities had initially hoped to integrate their mHealth programs into the primary care workflow, provider time constraints and the lack of reimbursement for helping with txt4health and Care4Life enrollment were perceived as hindering these efforts.

Despite these challenges, the Beacon Communities were able to involve care team members in promoting their mHealth programs. Practice coordinators in Southeast Michigan incorporated txt4health enrollment into patient check-in and check-out processes; additionally, diabetic patients participating in the Patient Health Navigator care management program, and those who were identified by the Emergency Department Diabetic Patient Identification program, were encouraged to enroll in txt4health.

The Utah Beacon team also interfaced with primary care clinics because they anticipated benefits to integrating the Care4Life pilot into patients’ existing care settings and because providers expressed interest in self-management support for patients outside of the clinic. Since the Utah Beacon offered pay-for-performance incentives to a subset of clinics based on diabetes care quality and outcomes, medical assistants were able to enroll patients in Care4Life as a strategy to reach hemoglobin A1c (HbA1c, a measure of blood sugar control) targets. However, in the absence of such payment incentives, the clinics may not have chosen this approach, as it required medical assistants to devote time they otherwise would have spent on patient care.

Another barrier to integration of mHealth into the care setting is that few existing mHealth tools have the capability to interface with the data management systems or EHRs used by clinicians to record patient encounter information [32]. Therefore, information collected as part of mHealth programs (via text or otherwise) like Care4Life or txt4health is unlikely to be recorded or viewed by providers in their primary documentation and clinical decision support systems, and therefore unlikely to be used to inform decisions at the point of care.

In cases where mHealth programs are able to send data to EHRs, providers have expressed concern about how to handle those data, raising questions of information accuracy and medical liability [33]. Given the variety and volume of data that providers are already struggling to process and manage as they adopt EHRs and other health IT tools, it is unclear whether or to what extent additional patient-generated information from mHealth programs will be integrated into the care process directly through these enabling technologies. Thus, while new reimbursement structures may facilitate integration of mHealth into the primary care workflow, further advances in device interoperability and data integration will also be necessary to achieve this objective.

## Evaluating Impact

The Beacon Communities are pioneers in deploying mHealth programs to achieve the triple aim of reduced costs, improved population health, and higher-quality care, and many stakeholders are anxious to see what impact these and similar initiatives have had. All three Beacon Communities are engaged in evaluation efforts, the results of which will be disseminated separately. While initial results are promising in terms of user satisfaction and self-reported behavior change, assessment of these mHealth interventions has proved particularly challenging. The domain of mHealth interventions is new and rapidly evolving, and standardized and robust evaluation methodologies are not yet widely available [32,34]. As a result, much of the existing literature focuses on the feasibility of deploying mHealth programs, rather than their impact on health outcomes; the little evidence available on the impact of mHealth is highly variable and often context specific [32,34].

As it happened, the Beacon Communities were offered the opportunity to launch the txt4health and Care4Life pilot programs more than 1 year into these 3-year efforts. Thus, while the Beacon grants initially allocated funding for robust program evaluations, the post-hoc funding re-allocated to txt4health and Care4Life included relatively few resources specifically for evaluation. As a result, the Beacon teams needed to take a pragmatic approach to evaluation and, in some cases, secure funding from other sources (eg, community partners).

The Beacons also faced an accelerated timeline, needing to complete the entire pilot (including program planning, development, implementation, deployment, evaluation, and close out) in less than 2 years. Given the aforementioned resource constraints and the relative dearth of evidence available at the time of launch regarding best practices for mHealth deployment, the Beacons worked to balance the desire for rigorous evaluations with the need to rapidly roll out the programs. In addition to the roll-out processes described above, the evaluation teams were charged with designing evaluation plans, obtaining Institutional Review Board (IRB) approval, recruiting active users, administering surveys, and collecting and analyzing the resulting data.

In the context of these constraints, the Beacons took different approaches to evaluating their specific programmatic objectives (Table 5). Primary data sources included txt4health and Care4Life system usage data, EHR data, and multimodal surveys offered online and via text, mail, or phone. From these sources, the evaluation teams gathered data to inform multiple outcomes of interest, including enrollment numbers, user demographics, user engagement (eg, number/frequency of texts responded to, duration in the program), clinical outcomes (eg, change in HbA1c), self-reported behavior (eg, medication adherence), patient activation, and user satisfaction. Patient activation is assessed with the Patient Activation Measure (PAM), a valid, highly reliable scale that reflects a developmental model of patient and consumer activation [35].

During data collection, the Beacon teams encountered limitations including attrition bias, sampling bias, non-response to surveys, and incomplete EHR data due to inconsistent primary care follow-up. For example, in Utah, the Care4Life team had planned to evaluate objective change in HbA1c using data from participating provider EHRs. However, these data were missing for many patients who did not come in for regular follow-up appointments as the Utah Beacon team had assumed they would; this greatly reduced the sample size available for analysis. In Crescent City, the txt4health team chose landline random digit dialing (RDD) as a survey method since it was less expensive than more robust methodologies (eg, mobile phone RDD, which requires additional screening to ensure numbers in the sample are active and local). Unfortunately, landline RDD may have introduced selection bias by oversampling populations that are more likely to use landlines (eg, older individuals) and undersampling those more likely to use mobile phones. Since the survey was intended to evaluate a mobile phone–based service, this bias may have important consequences for the validity of the results.

An additional challenge was the difficulty of isolating the impact of mHealth programs on health behaviors and outcomes. As was the case with many mHealth programs, txt4health and Care4Life were implemented in “real-world” settings rather than controlled research settings, in the midst of multiple Beacon Community initiatives aimed at improving diabetes care and outcomes. This context makes it difficult to control for external factors and tease out the impact of—or attribute observed outcomes to—the specific mHealth intervention.

Despite these limitations, useful insights may be gleaned from the available data sources and analyses. In addition to the initial results of self-reported behavior change, patient activation, and user satisfaction, correlations between particular demographic characteristics and enrollment and program usage data may reveal important information. For instance, these data may help determine which people are most likely to enroll in, engage with, and benefit from these programs; how and why they choose to use the programs; whether one-way or two-way messaging is more effective in driving behavior change; and whether certain characteristics correlate with higher likelihood of dropping out of the program [36].

In some cases, the limitations and biases associated with mHealth data sources and evaluation methodologies can be addressed, but generally at a cost. Those engaged in mHealth evaluation efforts must consider the costs and benefits, as well as the anticipated value and intended use of evaluation results. For example, if the evaluation is to be used to assess clinical impact, or to justify further significant resource expenditure to sustain and/or spread an mHealth program, then the value of anticipated outcomes may be worth the costs of rigor. Alternatively, programs that primarily focus on health education and public awareness may only need rigor sufficient to prove the value of the program to community partners and other local supporters and thus may be able to use lower-cost methodologies. Regardless, those embarking on mHealth interventions should carefully consider their evaluation and research aims from the outset, as well as the resources they have at their disposal to achieve their desired outcomes [32].

**Table 5:** Beacon Community evaluation strategies for txt4health and Care4Life.

mHealth Program (Beacon Community )	Program Component Being Evaluated	Outcome(s) of Interest	Evaluation Method(s)
**Txt4health (Crescent City)**			
	Social marketing campaign	Awareness of and support for txt4health	Cross-sectional pre- and post- campaign surveys (online and landline Random Digit Dialing)
	**User engagement**		
		# of users enrolled	Descriptive analysis of system-level usage data
		% of users completing diabetes risk assessment	
		Frequency of setting/achieving physical activity and weight loss goals	
		Patient Activation Measure (PAM) score	Patient Activation Measure (PAM) tool
	**User satisfaction**		
		User demographics	Multimodal survey (telephone, online, or mail)
		User perceptions of txt4health usability	
		Impact on user behavior	
		User satisfaction	
**Txt4health (Southeast Michigan)**			
	**User engagement**		
		# of users enrolled	Descriptive analysis of system-level usage data
		% of users completing diabetes risk assessment	
		Frequency of setting/achieving physical activity and weight loss goals	
		Patient Activation Measure (PAM) score	Patient Activation Measure (PAM) tool
	**User satisfaction**		
		User demographics	Multimodal survey (telephone, online, or mail)
		User perceptions of txt4health usability	
		Impact on user behavior	
		User satisfaction	
**Care4Life (Utah)**			
	Clinical outcomes	Change in HbA1c	Electronic health record review
	**User engagement**		
		Duration in the program	Descriptive analysis of system-level usage data
		# of text message replies with the program	
		Frequency of messages elected to receive	
	User satisfaction	User satisfaction	5-question text message-based survey at 90 days
			Client Satisfaction Questionnaire (CSQ-8) at 180 days

## Scaling and Sustaining mHealth Initiatives

As with the other interventions they implemented, the Beacon Communities launched these mHealth pilots in hopes that—if demonstrated to be effective at achieving their objectives—they would be sustained and eventually scaled to other populations and/or communities. However, developing long-term plans to sustain and scale these programs has proven challenging, and the future of the programs in some communities remains uncertain.

Only Crescent City is planning to expand txt4health statewide throughout Louisiana; in the meantime, participants in the New Orleans area can still enroll in the program. The Utah Beacon continues to recruit and enroll patients in Care4Life through the end of the Beacon Program in September 2013, but there are currently no plans to sustain or scale it beyond that time. In Southeast Michigan, all marketing and enrollment for txt4health concluded following the end of the year-long pilot. Despite the diverse and uncertain futures of these programs, the Beacon Communities identified several approaches that could facilitate the long-term sustainability of community-based mHealth programs: incorporating mHealth programs into the care setting, engaging payers, and leveraging community resources and organizations to reach target constituents.

As noted previously, incorporating mHealth into the clinical setting facilitates enrollment and thus, represents a promising sustainability strategy. However, changes to the payment structure that reward improvements in patient health are necessary to make mHealth and other health IT strategies sustainable. By participating in new payment models such as accountable care organizations and/or partnering with health plans, providers and staff could be reimbursed for activities that promote patient self-care including explaining mHealth programs, helping patients enroll, and reviewing patient data submitted via mHealth programs. The Beacons are also integrating txt4health into other proven diabetes prevention programs that are reimbursable activities, such as a YMCA diabetes prevention program in Crescent City and an Emergency Department Diabetic Patient Identification intervention in Southeast Michigan. This strategy supports sustainability efforts while enhancing diabetes prevention offerings to at-risk populations.

While these types of payment and care delivery reform efforts are becoming widespread, numerous barriers still hinder efforts to engage payers to support mHealth. For instance, in Michigan, one challenge stemmed from the numerous requirements and approvals required for marketing communications directed toward Medicaid beneficiaries. These requirements clearly applied to txt4health and required the Southeast Michigan Beacon Community to secure state approvals before payers could promote txt4health to Medicaid-insured individuals.

In all three Beacon Communities, building relationships with community organizations and leveraging local resources was critical to the success of their mHealth interventions. Building trust and social capital through these partnerships provided outreach channels to the organizations’ constituents, as well as sources of in-kind support (eg, creative development, sponsorship of campaign events), campaign design input, and in some cases, financial support.

For example, the Crescent City Beacon Community advisory group included many traditional public health partners (eg, state and local health departments, health associations) and several non-traditional private sector partners (eg, large health plans, employers, faith-based organizations, fraternity/sorority groups). These organizations were selected based on their reach and influence among targeted communities, and capacity to provide financial and in-kind support for public health initiatives. Maintaining effective communication and allowing advisory group members to help shape the program from project inception was a key to success. Establishing trust among key stakeholders who buy into a mutually beneficial concept can facilitate scaling and sustainability of grassroots activities and pilot programs.

## Conclusions

In the midst of widespread attention to mHealth as the “next big thing” in health care, the collective experience of these three communities provides insights into the practical challenges of implementing mHealth programs in the community setting. Beacon Communities encountered a number of barriers at each stage, including issues related to developing tailored, culturally competent messages; designing comprehensive outreach strategies; enrolling participants; engaging providers in mHealth programs; evaluating mHealth programs; and sustaining and scaling pilots. Ideally, others with an interest in implementing community-based mHealth programs will be able to apply these lessons learned to help anticipate and overcome potential challenges in their own initiatives.

The Beacon experiences also yielded important insights into what works. These factors were critical to the success of their mHealth programs and should be considered by other communities:

1. Identify community partners that are willing to engage with and support the program, and leverage their resources and community presence to design the program strategy and reach the target audience.
2. To the extent possible, design outreach, enrollment, and message content around the needs and perspectives of end users to increase program enrollment and engagement.
3. Anticipate that traditional marketing tactics may be insufficient to drive enrollment, and plan and budget additional staff time and resources for in-person engagement with the target audience to help drive enrollment.
4. To the extent feasible, bring care providers into the process—even if it means developing work-around solutions—to help them understand and promote mHealth as a tool to enhance patient care.
5. When planning the evaluation strategy, decide at the outset which aspects of the program will be critical to measure and which will not, to determine what stakeholders “need to know” versus what would be “nice to know”.
6. Last, share lessons learned with others to allow them to benefit from your experience.

A certain level of readiness is necessary for both providers and patients to begin to use cell phones as sources of and channels for sharing health information. This readiness may take time to develop. Much like the adoption curve for other technologies (eg, automated teller machines, online retail transactions), the use of mHealth may require time for market adoption and product improvement [36,37]. Working toward a culture of greater patient engagement in health and care will also further the potential impact of mHealth. And, as noted previously, framing and tailoring mHealth messages to target health beliefs and perceptions may enhance their impact on behavior change.

A primary take-away from the Beacon Community experiences with txt4health and Care4Life is that mHealth technology itself is not a “silver bullet”. As is increasingly evident in the adoption of many other health IT tools, the full value of mHealth will be realized only when attitudes, behaviors, and health care delivery also change.

## Multimedia Appendix 1

Voxiva, txt4health, and Care4Life.

## Abbreviations

ADA: American Diabetes Association
EHR: electronic health record
IRB: Institutional Review Board
NDEP: National Diabetes Education Program
RDD: random digit dialing
