# Supplementary material for: Considerations for Community-Based mHealth Initiatives: Insights From Three Beacon Communities
Source: J Med Internet Res. 2013 Oct 15;15(10):e221. doi: 10.2196/jmir.2803 (PMC3806518; doi:10.2196/jmir.2803)
Supplement: Supplementary file 1 [file jmir_v15i10e221_app1.pdf]

Multimedia Appendix 1. Voxiva, txt4health, and Care4Life.

The txt4health and care4Life programs were developed by Voxiva, a technology company that develops mHealth services across a broad range of health topics including maternal & child health, smoking cessation, and diabetes self-management. The Beacon Communities were the first to pilot txt4health, whereas Voxiva had previously adapted and pilot-tested Care4Life from a similar mHealth program originally launched in Mexico. Voxiva had no part in the preparation of this manuscript.

Since this pilot program, Txt4healthSM has evolved from a diabetes risk assessment tool to a broader health and wellness mobile health coaching service designed to help people take simple steps to lead healthy lives. Txt4health provides subscribers with reminders about important health exams, builds their knowledge about diabetes and other health topics with tips, quizzes, and resources and gives them opportunities to actively improve their health with personalized 30-day challenges.

For more information about Voxiva products and the availability of txt4health and Care4Life, see their respective websites:

- Voxiva
- Care4Life
- Txt4health
